# iTRAQ-based proteomic profiling reveals different protein expression between normal skin and hypertrophic scar tissue

**DOI:** 10.1186/s41038-015-0016-6

**Published:** 2015-08-27

**Authors:** Jianglin Tan, Weifeng He, Gaoxing Luo, Jun Wu

**Affiliations:** Institute of Burn Research, State Key Laboratory of Trauma, Burns and Combined Injuries, Chongqing Key Laboratory for Disease Proteomics, Southwest Hospital, Third Military Medical University, Chongqing, 400038 China

**Keywords:** iTRAQ, Hypertrophic scar, Proteomics

## Abstract

**Background:**

A hypertrophic scar is a unique fibrotic disease that only exists in humans. Despite advances in burn care and rehabilitation, as well as progress in the management during these decades, the hypertrophic scar remains hard to cure following surgical methods and drugs for treatment. In this study, we are looking forward to finding the multitude of possible traumatic mechanisms and the underlying molecular signal ways in the formation of the hypertrophic scar.

**Methods:**

We used isobaric tags for relative and absolute quantitation (iTRAQ) labeling technology, followed by high-throughput 2D LC-MS/MS, to determine relative quantitative differential proteins between the hypertrophic scar and normal skin tissue.

**Results:**

A total of 3166 proteins were identified with a high confidence (≥95 % confidence). And, a total of 89 proteins were identified as the differential proteins between the hypertrophic scar and normal skin, among which 41 proteins were up-regulated and 48 proteins were down-regulated in the hypertrophic scar. GO-Analysis indicated the up-regulated proteins were involved in extracellular matrix, whereas the down-regulated proteins were involved in dynamic junction and structural molecule activity.

**Conclusions:**

In our study, we demonstrate 89 proteins present differently in the hypertrophic scar compared to normal skin by iTRAQ technology, which might indicate the pathologic process of hypertrophic scar formation and guide us to propose new strategies against the hypertrophic scar.

## Background

A hypertrophic scar, a unique fibrotic disease in humans as no animals are known to form these lesions, presents as erythematous, firm, elevated plaques that remain confined to the area damaged by the initial injury [1]. Although it does not pose a health risk, some scars may be associated with pruritus, pain, disfigurement, disfunction, and psychological distress [2, 3]. Despite advances in burn care and rehabilitation, as well as progress in the management during these decades, the pathologic scar remains hard to cure following surgical methods and drugs for treatment [4, 5]. On this background, we are looking forward to finding the multitude of possible traumatic mechanisms and the underlying molecular signal ways in the formation of the hypertrophic scar.

In our previous studies, we have compared the global gene profiling from the normal skin and hypertrophic scar samples via cDNA microarray analysis [6]. The altered genes were related to proto-oncogenes, apoptosis, immune regulatory genes, cytoskeletal element, metabolism, and so forth. Besides the genomic approach, we further compared the protein profiles with a proteomic approach in this study. The isobaric tags for relative and absolute quantitation (iTRAQ) is a shotgun-based technique which allows the concurrent identification and relative quantification of hundreds of proteins in up to different biological samples in a single experiment [7]. With the iTRAQ approach, a large-scale evaluation of differential protein expression in the formation of the hypertrophic scar may be valuable for finding scar-related proteins or the potential target to control the hypertrophic scar.

In this article, we revealed the differential proteomics between the hypertrophic scar and normal skin tissues. From three patients, 3166 proteins were screened by iTRAQ. Forty-one up-regulated proteins were related to extracellular matrix, and 48 down-regulated proteins were involved in dynamic junction and structural molecule activity.

## Methods

### Tissue procurement and patient characteristics

The patient informed consent forms along with tissue procurement procedures were approved by the Ethic Committee of Southwest Hospital, Chongqing, China. All the hypertrophic scar patients were selected according to the Vancouver Scar Scale (VSS) ranging from a score of 10 to 13. The hypertrophic scar tissues and the normal skin tissues were obtained from the same patients who underwent orthopedic surgery at the Institute of Burn Research of Southwest Hospital. The tissues were frozen in liquid nitrogen immediately after surgical removal and stored at −80 °C till sample preparation.

### Reagents and chemicals

The chemical reagents acetonitrile, ethanol, methanol, acetone, ammonium formate (high-performance liquid chromatography (HPLC) grade), and trifluoroacetic acid (TFA) were obtained from Sigma Corporation (Sigma, USA) and Fisher Science Corporation (Thermo, USA). The ultrapure-grade water, utilized for the HPLC and subsequent tandem mass spectrometry (MS/MS) analysis procedures, was generated from the MilliQ (Millipore, USA)-type water. All iTRAQ reagents and buffers were obtained from Applied Biosystems (Applied Biosystems, Foster City, CA, USA).

### Protein extraction

The tissues were minced to pieces of approximately 2 mm in size. The pieces of tissues were grinded with a mortar and pestle in liquid nitrogen. The soluble protein was extracted according to the protocol in the Partial Mammalian Proteome Extraction Kit (Calbiochem, USA). A volume of 1 ml of extraction reagent 1, 5 μl of protease inhibitor cocktail, and 500 μl glass beads were added immediately into 250 mg of the tissue powder. The sample was vortexed thoroughly for 1 min. After addition with a volume of 4 μl of Benzonase® Nuclease (Novagen, USA), the sample was incubated at 4 °C for 30 min at 500 rpm. The solubilized sample was centrifuged at 15,000 rpm for 30 min at 4 °C after ultrasonication. Then the precipitate was disintegrated with 1 ml detergent containing 500 μl 1 % (*w*/*v*) sodium dodecyl sulfate (SDS), 100 μl 1 % (*v*/*v*) NP-40, and 400 μl 10 M urea. Subsequently, the sample was incubated at 4 °C for 30 min at 500 rpm. And, the protein of low solubility was extracted after the ultrasonication. The sample was centrifuged at 15,000 rpm for 30 min at 4 °C, and the protein in the supernatant material was subjected to eight times the volume of ice-cold acetone precipitation overnight before re-suspending into 0.1 % (*w*/*v*) SDS, 0.1 % (*v*/*v*) NP-40, and 1 M urea. The soluble protein and the low soluble protein were quantified by the BCA protein assay kit (Pierce, USA) and stored in aliquots at −80 °C until use.

### In-solution digestion

The low soluble protein aliquot containing 250 μg of total protein in 250 μl was reduced by the addition of 25 μl of 100 mM dithiothreitol (DTT; final concentration is 10 mM) followed by incubation at 56 °C for 1 h. The reduced cysteine was alkylated by the addition of 25 μl of 550 mM IAA (final concentration is 55 mM) (Fisher Science, USA) for 1 h at room temperature in a dark condition. Then the unreacted IAA residues were neutralized by the addition of 42.5 μl of 100 mM DTT (final concentration is 17 mM). The protein was then digested in a 1:50 trypsin/protein ratio at 37.5 °C overnight. The digesting reaction was stopped by adding 367.5 μl of 0.1 % (*v*/*v*) TFA. The peptide samples were then dried in a vacuum concentrator and re-suspended by the addition of 250 μl of 0.1 % (*v*/*v*) TFA.

### iTRAQ reagent label

Dried tryptic digests were desalted using a reverse-phase system. The peptide samples were delivered to a Luna C-18 column (1 × 100 mm, 5 μm, 100 Å; Waters Corporation, Milford, MA, USA) and eluted by 80 % acetonitrile. Subsequently, the samples were dried again in the vacuum concentrator and re-suspended by the iTRAQ Dissolution Buffer with vortex mixing for 1 h. One unit of each iTRAQ reagent label (defined as the amount needed to label 100 μg of protein) was thawed and reconstituted in 70 μl of ethanol, with vortex mixing for 1 min according to the manufacturer’s protocol. The samples were mixed with the iTRAQ reagent for 1 h in a dark condition. A volume of 0.1 % TFA nine times the sample volume was added into the samples. The resulting labeled peptide samples were then pooled and dried in a vacuum concentrator before chromatographic fractionation. The samples were iTRAQ labeled in duplicate.

### First-dimensional HPLC analyses

The peptides were separated using a recently developed two-dimensional liquid chromatographic method which employs high pH reversed-phase separation in the first dimension. The labeled mixed peptide samples derived from approximately 200 μg of total protein were re-suspended in 200 μl of HPLC buffer A and gradient fractionated on a X-Terra C-18 column (1 × 100 mm, 5 μm, 100 Å; Waters Corporation, Milford, MA, USA) with a constant flow rate of 150 μl/min. The buffer A consisted of 20 mM ammonium formate (pH 10), and buffer B (pH 10) consisted of 10 % of 20 mM ammonium formate and 90 % of acetonitrile. The 65-min gradient started with 1 % buffer B and 99 % buffer A, followed by 1 to 40 % buffer B2 for 60 min, then 90 % buffer B2 for 5 min. The chromatogram was monitored through a UV detector. The UV wavelengths were set at 214 nm. Fractions were collected every minute and later were pooled together according to the variations in peak intensity. Pooled fractions were dried in a vacuum concentrator and stored at −80 °C until MS/MS analysis.

### Second-dimensional liquid chromatography-electrospray ionization-tandem mass spectrometry analysis

As described previously by Dwivedi et al. [8], a splitless nano-flow Tempo LC system (Eksigent, Dublin, CA, USA) with 20 μl sample (each dried labeled peptide fraction was re-dissolved in 25 μl of eluent A containing 0.1 % formic acid in 2 % acetonitrile) injection via a PepMap100 trap column (0.3× 5 mm, 5 μm, 100 Å; Dionex Corporation, Sunnyvale, CA, USA) and a 100 μm × 150 mm analytical column packed with 5-μm Luna C18(2) (Phenomenex, Torrance, CA, USA) was used in the second-dimension separation prior to tandem MS (MS/MS) analysis. Both eluents A (2 % acetonitrile in water) and B (98 % acetonitrile) contained 0.1 % formic acid as an ion-pairing modifier. A 0.44 % acetonitrile per minute linear gradient (0 to 35 % B in 80 min, 500 nl/min) was used for peptide elution, followed by a 5-min wash with 80 % B.

A QStar Elite QqTOF mass spectrometer (Applied Biosystems) was used in standard MS/MS data-dependent acquisition mode with a nano-electrospray ionization source. Survey MS spectra were collected (m/z 400 to 1500) for 1 s followed by three MS/MS measurements on the most intense parent ions (80 counts/s threshold, +2 to +4 charge state, and m/z 100 to 1500 mass range for MS/MS), using the manufacturer’s “smart exit” and “iTRAQ” settings. The “smart exit” option is a standard feature of the QStar Elite instrument, which runs under control of Analyst QS 2.0 software. It allows termination of MS/MS spectrum collection when preset values of peak intensity (quality of the spectra) are reached. In this way, the instrument spends less time for MS/MS acquisition of abundant species and improves chances for detection of low-abundant ones. Spectral quality setting 5 (whole scale 1–20) was used throughout the experiments. Predefined “iTRAQ” settings adjust (increase) collisional energy to maximize the intensity of reporter ions (114, 115, 116, 117 Da) in MS/MS spectra. Individual Wiff files generated after QStar Elite analysis were converted to mascot generic file (MGF) format using Mascot.dll script in Analyst QS 2.0. Following this conversion, MGF files of individual fractions were combined into one using a merging script [9].

### Database search and protein identification

The MS/MS data were analyzed using ProteinPilot software version 2.0.1 (Applied Biosystems/MDS Sciex, Concord, ON, Canada). The search parameters were complete modifications of Cys alkylation with IAA, and inbuilt iTRAQ analysis residue modifications settings were on. Those protein candidates with greater than or equal to 95 % identification confidence were used for further analysis [8]. The annotation of protein cellular localization and biological function was performed using PPI network (Ingenuity Systems, Inc., Redwood City, CA, USA).

## Results

### Overview of proteomic changes in the hypertrophic scar comparing normal skin

A detailed analysis of the proteomic changes was performed using iTRAQ labeling. Reporters with masses of 114 and 115 were used to separately label biological replicates of normal skin, and reporters with masses of 116 and 117 were used for replicates of the hypertrophic scar. The four isobaric tag samples were mixed and analyzed by 2D-MS/MS. Relative protein levels were determined by comparing peak intensities of the four reporter ions released from each pool of purified, labeled peptides. We identified 3166 distinct proteins. Eighty-nine proteins were identified with a high confidence to up- or down-regulate in the hypertrophic scar (Table 1).

**Table 1:** A list of up-regulated proteins (*n* = 20) and down-regulated proteins (*n* = 27) identified by iTRAQ labeling combined with 2D LC-MS/MS

Accession	Name	Average fold change
ENSP00000222271	Cartilage oligomeric matrix protein	5.841842
ENSP00000347041	Fibromodulin	5.594914
ENSP00000364694	Asporin	4.344845
ENSP00000341170	Pleiotrophin	3.243324
ENSP00000359153	Collagen, type XII, alpha 1	3.201809
ENSP00000362122	Tenomodulin	3.182895
ENSP00000300026	Peptidylprolyl isomerase B (cyclophilin B)	3.071857
ENSP00000360882	Collagen, type V, alpha 1	2.865116
ENSP00000260356	Thrombospondin 1	2.668636
ENSP00000215909	Lectin, galactoside-binding, soluble, 1	2.555282
ENSP00000216336	Cathepsin G	2.541028
ENSP00000350894	Serpin peptidase inhibitor, clade H (heat shock protein 47), member 1	2.502299
ENSP00000346839	Fibronectin 1	2.156667
ENSP00000265131	Tenascin C	2.12325
ENSP00000225964	Collagen, type I, alpha 1	1.998597
ENSP00000296511	Annexin A5	1.912369
ENSP00000254722	Serpin peptidase inhibitor, clade F, member 1	1.89519
ENSP00000297268	Collagen, type I, alpha 2	1.88893
ENSP00000304408	Collagen, type III, alpha 1	1.855389
ENSP00000364711	Osteoglycin	1.713209
ENSP00000407788	Inner membrane protein, mitochondrial (mitofilin)	0.69212
ENSP00000327070	Malate dehydrogenase 2, NAD (mitochondrial)	0.685238
ENSP00000379158	Calpastatin	0.672655
ENSP00000334983	Pyruvate kinase, muscle 2	0.626574
ENSP00000348965	Dynein, cytoplasmic 1, heavy chain 1	0.617843
ENSP00000200181	Integrin, beta 4	0.616907
ENSP00000417773	Transketolase	0.615061
ENSP00000344504	H1 histone family, member 0	0.587647
ENSP00000347190	Catenin (cadherin-associated protein), alpha 1, 102 kDa	0.572509
ENSP00000343129	Tripartite motif-containing 29	0.571671
ENSP00000406273	Myosin, heavy chain 14, non-muscle	0.559878
ENSP00000375938	Tubulin, alpha 4a	0.54832
ENSP00000269576	Keratin 10	0.530142
ENSP00000331678	Plakophilin 3	0.521494
ENSP00000300036	Myosin, heavy chain 11, smooth muscle	0.519123
ENSP00000377508	Junction plakoglobin	0.49407
ENSP00000353608	Desmocollin 3	0.490851
ENSP00000301607	Envoplakin	0.487905
ENSP00000377550	Keratin 15	0.473652
ENSP00000420296	S100 calcium binding protein A14	0.418854
ENSP00000252242	Keratin 5	0.390976
ENSP00000295597	Plakophilin 1 (ectodermal dysplasia/skin fragility syndrome)	0.35161
ENSP00000290158	Karyopherin (importin) beta 1	0.335195
ENSP00000363071	Desmin	0.330774
ENSP00000369129	Desmoplakin	0.326555

### Proteomic analysis of the differential up-regulated expression proteins

**Fig. 1:**
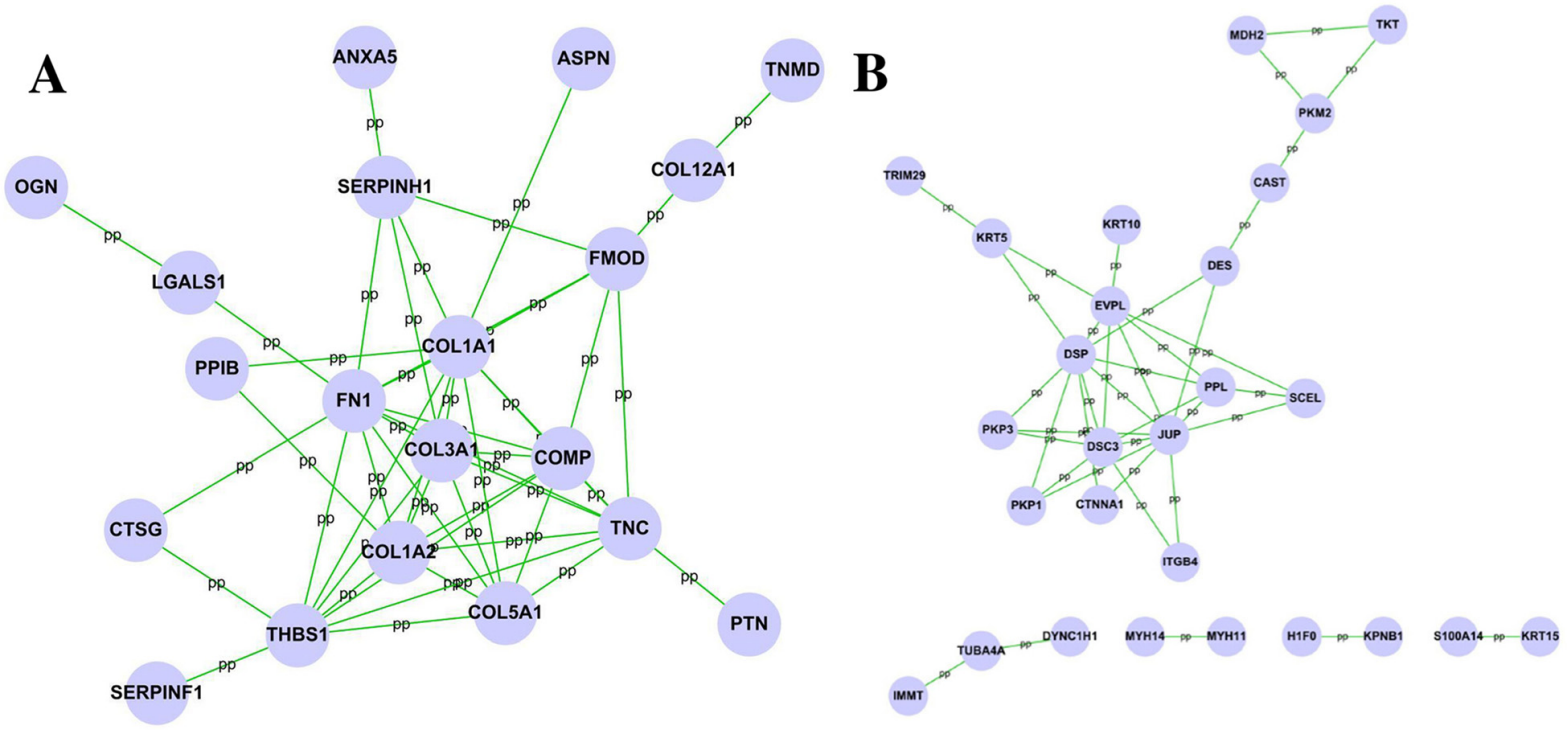
Establishment of protein-protein interaction networks for scar/skin tissue differential proteins. The protein-protein interaction networks were constructed by cytoscape software according the HPRD. Proteins are represented with *grey nodes*, and interactions are represented with *edges*. **a** Scar significantly up-regulated PPI network was constructed, 20 of 41 scar-up-regulated proteins were included in the network. **b** Scar significantly down-regulated PPI network was constructed, 27 of 48 scar-up-regulated proteins were included in the network

**Fig. 2:**
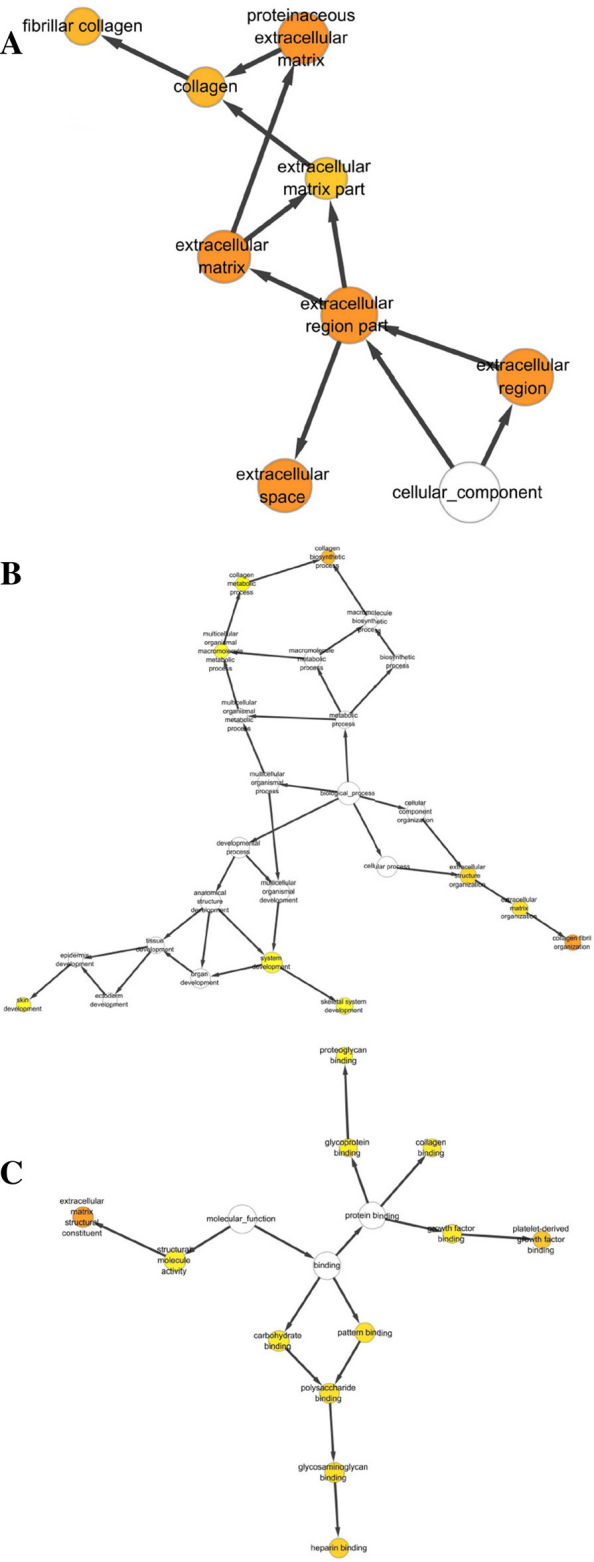
Scar-up-regulated PPI network was analyzed by BinGO plugin in cytoscape software. **a** Biological process categories map. Map of biological process categories associated with the scar-up-regulated PPI network. **b** Cellular component map. Map of the cellular component associated with the scar-up-regulated PPI network. **c**. Molecular function map. Map of molecular function associated with scar-up-regulated PPI network. *Darker nodes* refer to the significant ontologies of the dataset. The size is proportional to the number of genes that participate in that molecular function

**Fig. 3:**
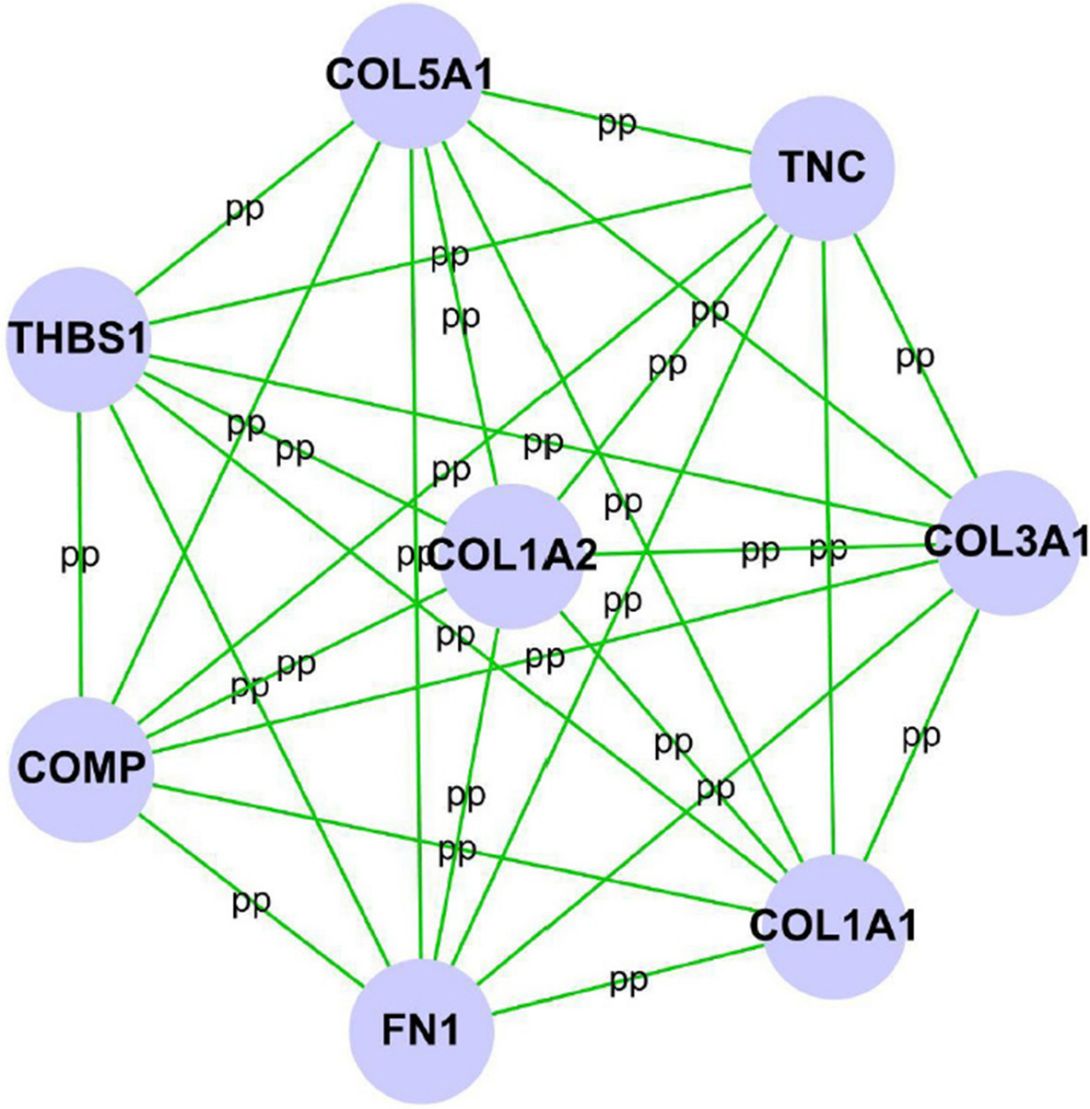
Cluster analysis of the scar-up-regulated PPI network by MCODE plugin in cytoscape software. We identified eight genes belonging to the highest cluster, i.e., the leader genes: COL1A1, COL1A2, COL3A1, COL5A1, COMP, FN1, THBS1, and TNC

Among these differential expressed proteins, 20 of 41 up-regulated proteins (Fig. 1a) were analyzed by cytoscape software to indicate a diversified functional network. BinGO enrichment demonstrated that 20 up-regulated protein categories were enriched in the biological process (Fig. 2a), cellular component (Fig. 2b), and molecular function (Fig. 2c) categories. Among these proteins, extracellular matrices such as collagen, type I, alpha 1 (COL1A1); collagen, type I, alpha 2 (COL1A2); collagen, type III, alpha 1 (COL3A1); collagen, type V, alpha 1 (COL5A1), cartilage oligomeric matrix protein (COMP), fibronectin 1 (FN1), thrombospondin 1 (THBS1), and tenascin C (TNC) were identified to be the highest cluster (Fig. 3).

### Proteomic analysis of the differential down-regulated expression proteins

**Fig. 4:**
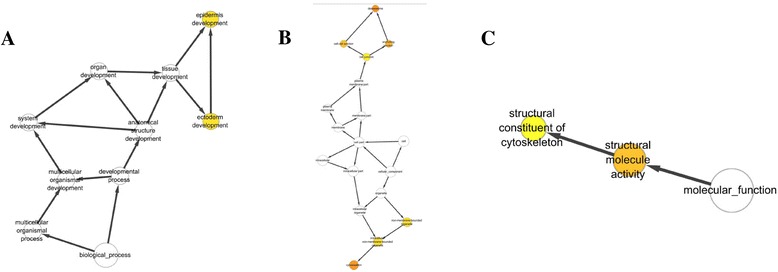
The scar-down-regulated PPI network was analyzed by BinGO plugin in cytoscape software. **a** Biological process categories map. Map of biological process categories associated with the scar-down-regulated PPI network. **b** Cellular component map. Map of cellular component associated with scar-down-regulated PPI network. **c**. Molecular function map. Map of molecular function associated with scar-down-regulated PPI network. *Darker nodes* refer to the significant ontologies of the dataset. The size is proportional to the number of genes that participate in that molecular function

**Fig. 5:**
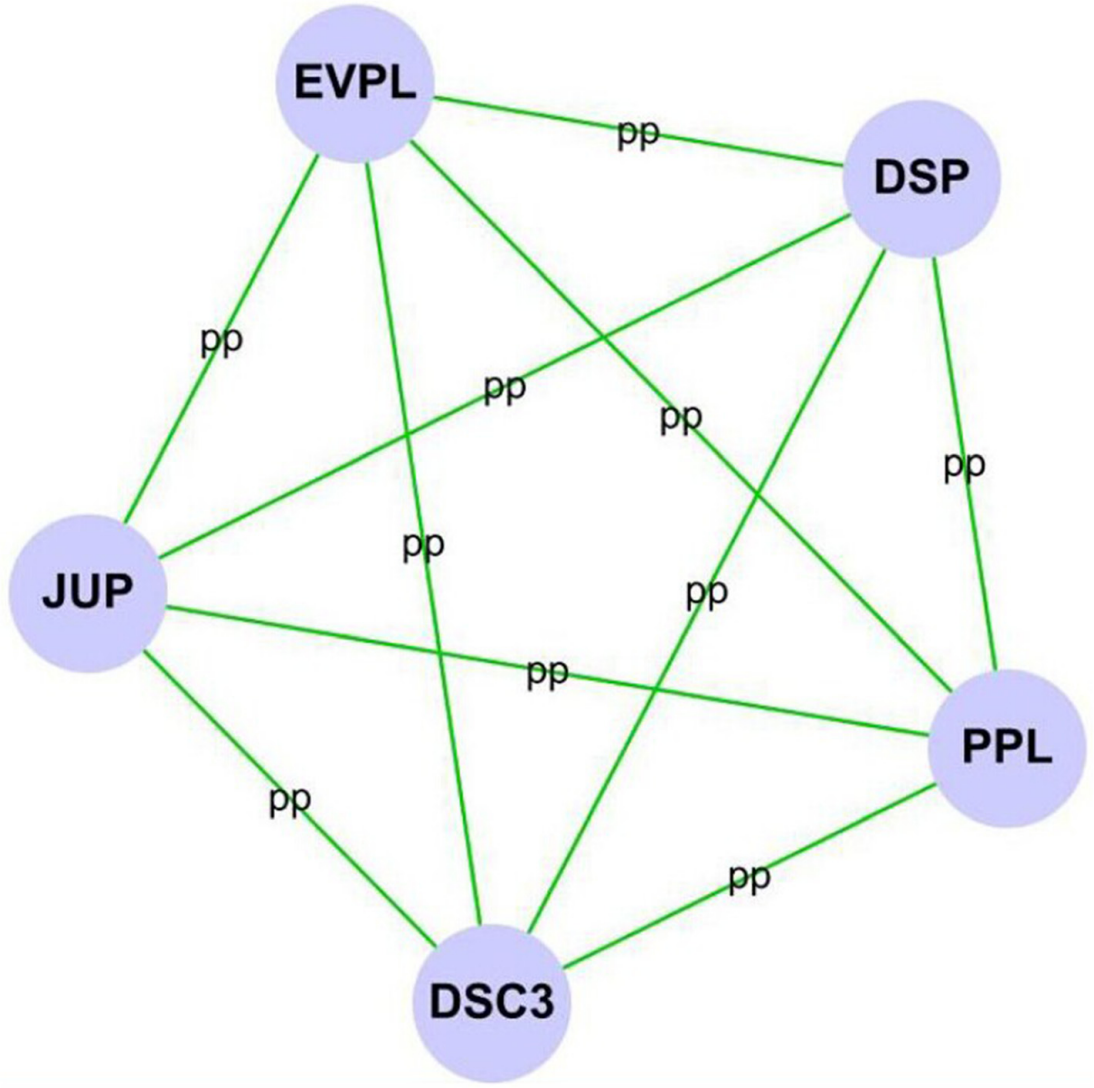
Cluster analysis of the scar-down-regulated PPI network by MCODE plugin in cytoscape software. We identified five genes belonging to the highest cluster, i.e., the leader genes: DSC3, DSP, EVPL, JUP, and PPL

Twenty-seven of 48 down-regulated proteins were analyzed by cytoscape software to indicate a functional network (Fig. 1b). A further 27 down-regulated proteins were involved in the biological process (Fig. 4a), cellular component (Fig. 4b), and molecular function (Fig. 4c) categories. And five proteins including desmocollin 3 (DSC3), desmoplakin (DSP), envoplakin (EVPL), junction plakoglobin (JUP), and periplakin (PPL) were related to the highest cluster (Fig. 5).

## Discussion

In this study, we use the iTRAQ method to screen the differential proteins potentially involved in the hypertrophic scar compared to normal skin. Using 2D-MS/MS followed by GO-Analysis, we demonstrate 89 proteins with a high annotation confidence (≥95 %) present differently in the hypertrophic scar. Among the differential proteins, 41 proteins increased and 48 proteins decreased in the hypertrophic scar. Consistent with previous studies, the up-regulated proteins such as COL1A1, COL1A2, COL3A1, COL5A1, COMP, FN1, THBS1, and TNC were involved in extracellular matrix production, myofibroblast contractility, and response to mechanical stress [10–13]. Interestingly, most of the down-regulated proteins, including DSC3, DSP, EVPL, JUP, and PPL, were specific to the epidermis and involved in the cell junction [14–18].

Some of the differentially expressed candidates identified by iTRAQ have previously been associated with hypertrophic scar formation. Of the up-regulated proteins, increased COL1A1, COL1A2, COL3A1, FN1, and TNC expression have been reported in the hypertrophic scar. COMP plays a role as a matrix deposition promoter in the keloid (the special fibrotic skin disease) [19]. It has been reported that THBS1 may modulate keloid formation through up-regulation of the matrix plasminogen/plasmin system [20]. However, no data revealed the potential effects of COMP and THBS1 on the hypertrophic scar formation. Our work for the first time found COMP and THBS1 were up-regulated in the hypertrophic scar, which might be attributed to a new therapeutic target.

## Conclusions

In summary, the iTRAQ analyses followed by the high-throughput 2D LC-MS/MS in our study for the first time screened protein expression of the hypertrophic scar and normal skin tissue on a large scale from the same patients. Some of the screened proteins in our study have been reported in previous researches. However, some of the up-regulated proteins such as COMP and THBS1 and the down-regulated proteins could indicate that the pathologic process of hypertrophic scar formation which might guide us to propose new strategies against the hypertrophic scar.
